# Family identity as cultural capital: intergenerational solidarity and public health compliance in pandemic governance

**DOI:** 10.3389/fpubh.2026.1873402

**Published:** 2026-07-22

**Authors:** Yang Yang, Yuejun Cui, Guoliang Liu, Jianing Duan

**Affiliations:** 1 School of Economics, Beijing Wuzi University, Beijing, China; 2 School of International Economics, China Foreign Affairs University, Beijing, China

**Keywords:** cultural capital, family identity, game theory, health equity, intergenerational solidarity, public health compliance, social capital, sustainable health governance

## Abstract

**Introduction:**

Sustainable health governance requires moving beyond crisis response to strategic design that integrates cultural resources. However, the role of family-based cultural capital in shaping public health compliance remains underexplored.

**Methods:**

We develop a dynamic game-theoretic model of intra-household compliance and a two-period macro-level policy game. The model incorporates intergenerational solidarity theory and conceptualizes Confucian family identity as a form of cultural capital.

**Results:**

Our model shows that family cultural capital can resolve the household prisoner’s dilemma, transforming non-compliance into a cooperative equilibrium when normative and functional solidarity are strong. At the macro level, the government’s optimal investment in family cultural capital depends on a threshold condition: Type-I (high compliance, sustainable) equilibrium occurs when short-term economic pressure (D) is below a culturally-determined threshold; Type-II (low compliance, fragile) equilibrium occurs when D exceeds this threshold.

**Discussion:**

We derive four policy design principles: strengthening normative solidarity through cultural alignment, securing fiscal space to support family caregivers, investing in intergenerational solidarity as social infrastructure, and managing global interdependence to avoid the “high-return trap.” Our framework offers a culturally intelligent roadmap for designing resilient, equitable health governance systems.

## 1 Introduction

The 21st century’s landscape of frequent public health emergencies—from SARS and H1N1 to MERS, Ebola, and COVID-19—has evolved into a persistent stress test for global socio-economic systems and governance models (1). These crises expose a critical dilemma in global health security: the tension between the need for swift, collective action and the diverse cultural, political, and economic contexts in which such action must be implemented (2). While the International Health Regulations (IHR) provide a legal framework for coordination, their effectiveness ultimately depends on national implementation and public adherence (3). This implementation gap highlights a central challenge for sustainability science and health policy: how to design health governance systems that are not only effective in suppressing pathogens but also resilient, equitable, and socially sustainable across different cultural settings (4).

Recent research in health economics and management has increasingly recognized the importance of intergenerational solidarity and social capital as critical determinants of health outcomes and health system resilience. For instance, studies have shown that intergenerational contact and support networks significantly influence health equity among older adults, particularly during public health crises when formal care systems are strained (5). However, the role of cultural capital—specifically, deeply embedded family-based ethical systems—in shaping public health compliance remains underexplored. This gap is particularly salient in contexts where family structures serve as the primary locus of care, support, and social regulation.

Drawing on intergenerational solidarity theory (6) and the concept of social capital (7), we approach this challenge by conceptualizing Confucian family identity as a form of cultural capital that can be strategically leveraged to enhance public health compliance. Intergenerational solidarity theory identifies multiple dimensions—structural, associational, functional, normative, and affectional—through which family relationships influence individual behavior and well-being. Among these, normative solidarity (shared values and obligations) and functional solidarity (exchange of resources and support) are particularly relevant for understanding compliance with public health mandates during crises. When individuals internalize family-based norms of care and protection, they are more likely to perceive compliance not merely as a personal burden but as an expression of filial duty and family responsibility.

Current scholarly responses to the compliance challenge cluster in three domains, each with distinct limitations. First, retrospective policy analyses meticulously document specific national responses, often highlighting their context-dependent nature. Second, comparative studies of governance models dissect how historical legacies forge distinct “biosecurity states” (8, 9). Third, a growing literature applies public administration and political economy lenses to evaluate governance performance during crises (10, 37). Despite their value, these streams often fail to theorize how cultural systems—particularly family-based ethical frameworks—actively mediate and can be strategically engaged to shape the negotiation between state mandates and citizen behavior. Moreover, they rarely examine how intergenerational solidarity and family cultural capital can be harnessed to promote health equity and sustainable care arrangements.

A critical theoretical and practical gap persists: we lack a robust framework that explains and operationalizes how certain policy paradigms emerge as socially legitimate and garner high compliance within specific cultural milieus, while others provoke resistance or failure. This paper addresses this gap by proposing that sustainable health governance requires moving from a paradigm of imposition to one of strategic cultural negotiation. We argue that public compliance is brokered primarily within the arena of family-based cultural capital—especially culturally specific norms of intergenerational obligation and identity. These norms are not merely background conditions; they are actionable policy levers for enhancing health system resilience and reducing health inequalities.

Our investigation focuses on family identity—a Confucian-derived norm where social validation and self-worth are fundamentally tied to fulfilling familial roles and hierarchies—as a potent form of cultural capital and a potential policy lever for promoting intergenerational solidarity. We ask: How can sustainable health governance systems be designed to proactively leverage deep-seated family-based ethical frameworks to secure public compliance during protracted crises, while simultaneously promoting health equity across different household types?

To answer this, we develop a formal game-theoretic model that theorizes pandemic control as a strategic negotiation process between state policies and culturally embedded family interests. Unlike prior studies that treat culture as a static background variable, our model endogenizes family identity as a modifiable parameter that shapes intra-household incentives and compliance equilibria. We identify the conditions under which family cultural capital can be effectively leveraged—specifically, the role of state fiscal capacity in sustaining cultural-institutional alignment and the vulnerability of this mechanism to short-term economic pressures.

Our analysis makes three contributions to the health economics and management literature. First, we extend research on intergenerational solidarity by demonstrating how family-based cultural capital can be strategically activated to enhance public health compliance, thereby contributing to a more nuanced understanding of potential health equity implications, while leaving full formalization for future work. Second, we advance social capital theory by specifying the mechanisms—normative and functional solidarity—through which family identity translates into collective action in crisis contexts. Third, we provide actionable policy recommendations for integrating family cultural resources into formal health management frameworks, including differentiated strategies for supporting family caregivers and reducing health inequalities across household types. By bridging insights from intergenerational solidarity theory, social capital, and game theory, we offer a culturally intelligent roadmap for designing resilient, equitable, and legitimate health governance systems for future pandemics.

## 2 Literature review and conceptual framework

### 2.1 Sustainable health governance: from crisis response to strategic design

The concept of sustainable health governance transcends the technical goal of disease containment. It aligns with the Sustainable Development Goals’ emphasis on building resilient, equitable, and sustainable health systems. Scholars define it as the ongoing process of creating and reinforcing the institutions, rules, and norms that enable societies to effectively manage health threats while maintaining economic vitality, social equity, and political legitimacy over the long term (11, 12). Key pillars include resilience (the capacity to absorb, adapt, and transform in response to shocks), legitimacy (public perception that authority is appropriately exercised), and equity (fair distribution of burdens and benefits) (13, 14).

Recent research in health economics and management has increasingly emphasized the role of intergenerational solidarity and social capital as critical determinants of health system resilience. For instance, Vogel et al. (5) demonstrate that sustainable intergenerational contact patterns significantly influence health equity among older adults, particularly during public health crises when formal care systems are strained. This highlights the need to understand how family-based relationships and obligations can be leveraged as governance resources.

### 2.2 The compliance problem: a core challenge of governance design

Securing public compliance with intrusive health measures (e.g., lockdowns, isolation, and vaccination) is a classic collective action problem and a central test of governance effectiveness. Traditional public health models often rely on rationalist assumptions, expecting compliance based on the provision of information and perceived personal risk (15). However, empirical evidence consistently shows that compliance is shaped less by individual risk calculus and more by social norms, trust in institutions, and perceived fairness (16, 17). This situates the compliance problem squarely within the challenge of governance design, where the legitimacy of state action is continuously contested and validated by citizens (18).

From a health economics perspective, compliance can be understood as a resource allocation decision at the household level. Individuals (trade off) the private costs of compliance (e.g., lost income, social isolation) against the collective benefits of disease containment. When family-based cultural capital is strong, this cost–benefit calculus shifts, as non-compliance imposes not only potential legal penalties but also significant social and psychological costs within the family unit.

### 2.3 Intergenerational solidarity and family cultural capital

Defining cultural capital. Following Bourdieu (19), we define cultural capital as the embodied dispositions, knowledge, skills, and legitimate cultural competences that individuals acquire through socialization and education. Bourdieu distinguishes three forms: embodied (e.g., language, manners, taste), objectified (e.g., books, instruments), and institutionalized (e.g., educational credentials). In the context of public health compliance, the most relevant form is embodied cultural capital—specifically, internalized norms of family responsibility, health literacy, and the disposition to defer to expert authority during crises. This cultural capital shapes individuals’ cost–benefit calculus by altering the psychological and social utility of compliance.

Distinction from social capital. Cultural capital is conceptually distinct from social capital, which Bourdieu (19) defines as resources based on network membership and mutual acquaintance, and which Putnam (7) operationalizes as trust, reciprocity, and civic engagement. While social capital facilitates collective action through relational channels (e.g., “I comply because my neighbors do”), cultural capital operates through internalized dispositions (e.g., “I comply because I understand the scientific rationale and it aligns with my family values”). In our model, family identity functions primarily as cultural capital—it shapes preferences and internalized costs—rather than as social capital, though the two may co-occur and reinforce each other. We focus on cultural capital because it is the mechanism through which government narratives directly alter individual utility, a channel that remains undertheorized in the social capital literature.

Intergenerational solidarity theory provides a powerful lens for understanding how family relationships shape individual behavior and well-being. Bengtson and Roberts (6) conceptualize intergenerational solidarity through six dimensions: (1) associational solidarity (frequency of interaction), (2) affectual solidarity (emotional closeness), (3) consensual solidarity (agreement on values), (4) functional solidarity (exchange of resources and support), (5) normative solidarity (strength of commitment to familial obligations), and (6) structural solidarity (opportunities for interaction created by geographic proximity).

Among these dimensions, normative solidarity and functional solidarity are particularly relevant for understanding public health compliance during crises. Normative solidarity refers to the internalized sense of obligation to care for and protect family members. When individuals internalize strong family-based norms, compliance with public health mandates becomes framed not as a personal burden but as an expression of filial duty and family responsibility. Functional solidarity, which encompasses the exchange of material and instrumental support, provides the practical mechanisms through which families enforce compliance—for example, through monitoring, resource pooling, and caregiving arrangements.

This family-based solidarity can be understood as a form of cultural capital (19), which operates alongside—but is analytically distinct from—social capital (7). While social capital emphasizes networks and trust as relational resources, cultural capital emphasizes internalized dispositions and legitimate competences. Social capital theory emphasizes that networks of reciprocal relationships generate trust, cooperation, and collective action. In the context of public health, family cultural capital functions as a informal governance resource that can supplement formal state mechanisms. When family solidarity is strong, the costs of monitoring and enforcement are partially socialized within the household, reducing the burden on state institutions and enhancing overall compliance.

### 2.4 Confucian family identity as cultural capital

The Confucian concept of family identity represents a particularly salient form of cultural capital in East Asian societies. Historians and sociologists have detailed how filial piety structures intergenerational support, resource pooling, and moral authority within East Asian families (20). Economists have modeled this as an implicit “intergenerational contract,” where care for elders is exchanged for inheritance and social status (21). This contract reduces transaction costs within the family and can act as a social safety net (22).

Crucially, Confucian family identity embodies both normative solidarity (the moral obligation to protect family members) and functional solidarity (the practical exchange of care and resources across generations). The Five Constants of Confucian ethics—Ren (benevolence), Yi (righteousness), Li (propriety), Zhi (wisdom), and Xin (trustworthiness)—provide a comprehensive normative framework that prioritizes social harmony and long-term orientation (38). This framework cultivates a stakeholder-centric worldview that contrasts with short-term individualistic profit maximization paradigms (39).

In crisis contexts, the family unit becomes a critical node for implementing or resisting state policy. Research on disasters in East Asia shows that family-centric mobilization can significantly amplify or dampen the effectiveness of official directives (23). However, formal analysis of the strategic interaction between this micro-level family institution and macro-level state health policy remains under-theorized. Specifically, existing research has not systematically examined how family cultural capital can be strategically activated to enhance public health compliance while simultaneously promoting health equity across different household types.

### 2.5 Game theory: formalizing strategic negotiation

Game theory offers a precise language for modeling strategic interactions where outcomes depend on the choices of multiple actors. It has been effectively used to study the emergence and stability of social norms (15) and compliance in public goods games (24). Applications to public health include modeling vaccination decisions (25) and social distancing (26).

However, most models treat preferences as exogenous and individualistic. Fewer have integrated endogenous, culturally-shaped preferences—like the utility derived from fulfilling familial duties—into the payoff structure of public health compliance games [for an exception in a different context, see (27)]. This gap presents an opportunity to formally model how family cultural capital operationalizes compliance within a strategic framework. By treating family identity as a parameter that shapes intra-household payoff structures, game theory can illuminate the conditions under which cultural resources effectively promote collective action.

### 2.6 Related literature and theoretical positioning

We extend our review to three additional literatures that inform our framework but have not been systematically integrated in prior work on public health compliance.

Game-theoretic models of intergenerational healthcare. Recent work has examined intergenerational healthcare as a strategic interaction (28, 29). However, both models treat preferences as fixed and focus on long-term care rather than pandemic compliance. Neither considers cultural capital as a preference-shifter nor endogenizes the government’s choice of cultural narratives.

Empirical work on cultural capital and COVID-19 compliance. Studies measuring cultural capital via educational attainment or health literacy have found robust correlations with mask-wearing, distancing, and vaccination (30, 31). This literature documents the fact of the relationship but does not explain the mechanism. Our model provides a micro-foundation for this relationship.

Identity economics: Akerlof and Kranton (27) introduced identity into economic analysis, with extensions to education (32), organizational economics (33), and political economy (34, 35). However, most models treat identity prescriptions as exogenous. Our contribution is to endogenize the government’s choice of cultural narratives as a policy lever.

Explicit contribution statement. Relative to these literatures: (i) we introduce cultural capital as a preference-shifter into intergenerational healthcare games; (ii) we formalize the mechanism underlying empirical cultural capital–compliance correlations; (iii) we model the government as an active identity entrepreneur, endogenizing cultural narrative choice.

Transparency note: A preliminary version of this research appeared as a student working paper in Horizon Academic (2023). We specify three substantive extensions that distinguish the current manuscript from the earlier version: (i) a fully specified game-theoretic model with complete government objective function, explicit threshold dynamics, and step-by-step derivations; (ii) systematic integration of intergenerational solidarity theory and a rigorous conceptual distinction between normative and functional solidarity; (iii) revised empirical illustrations reframed as plausibility probes with explicit discussion of health equity. We also note that the 2023 version is not peer-reviewed and served only as a preliminary draft, and that no data or results are reused without reanalysis.

### 2.7 Conceptual synthesis: a framework for leveraging family cultural capital

Synthesizing the above, we posit that sustainable health governance during a pandemic is an outcome of a strategic negotiation process shaped by intergenerational solidarity and family cultural capital.

The State employs formal policy levers (stringency, fiscal support, communication). The Citizen/Family operates within a cultural script—family identity—that defines internalized costs, benefits, and social sanctions. This family identity functions as cultural capital that can be strategically activated to enhance compliance and promote health equity.

The Negotiation Arena is where formal policy intersects with family-based solidarity. The effectiveness of policy levers is contingent upon their alignment with existing patterns of normative and functional solidarity within families. When alignment is achieved, the interaction produces outcomes like compliance, resistance, or adaptive cooperation.

Crucially, the success of this negotiation depends on two conditions. First, state fiscal capacity (*τ*) must be sufficient to sustain cultural-institutional alignment—for example, through material support for family caregivers. Second, short-term economic pressures (*D*) can undermine compliance by distorting incentive structures, creating a trade-off between immediate economic reopening and long-term health resilience.

**Figure 1:**
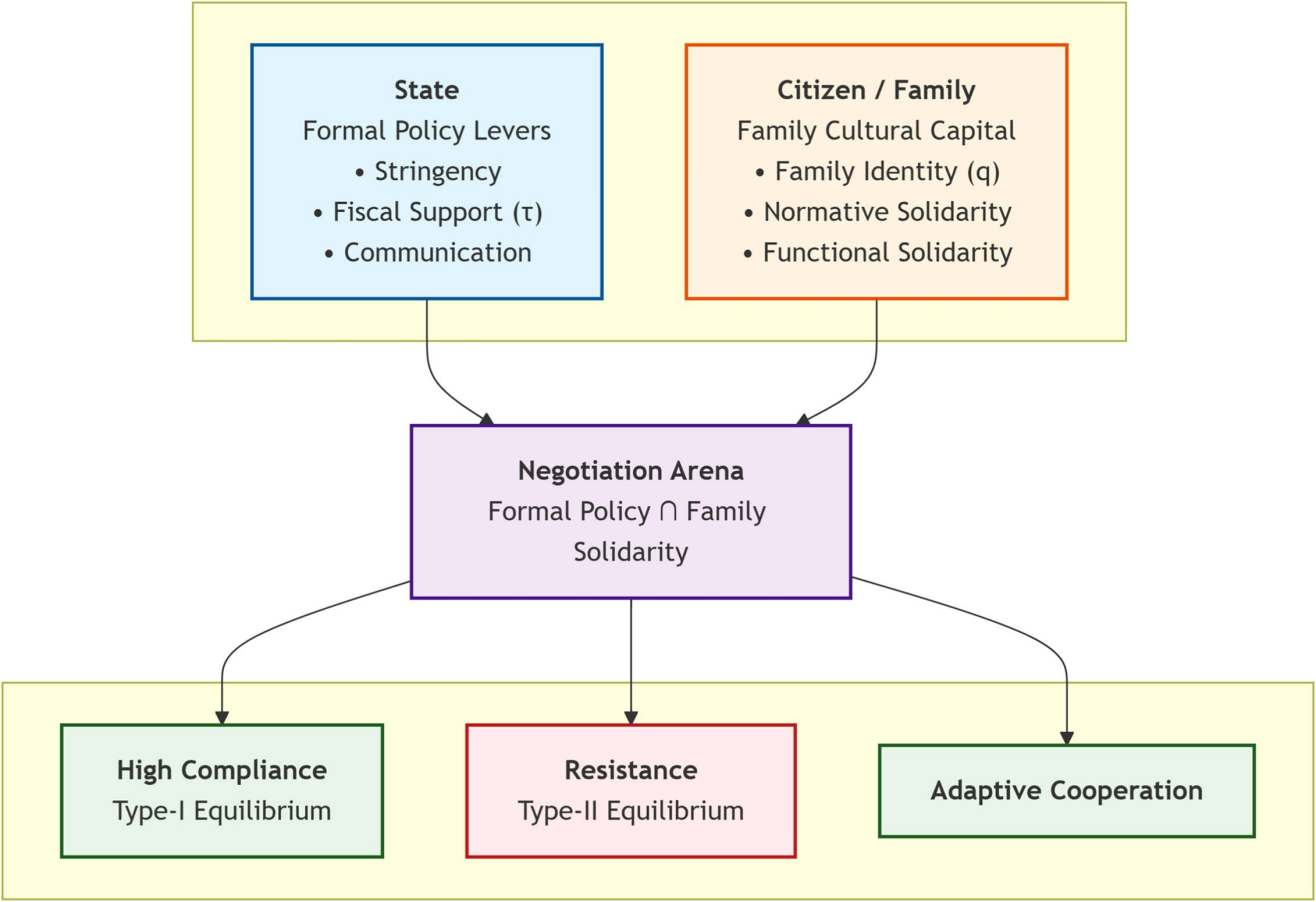
Conceptual framework.

Our game-theoretic model formalizes this strategic negotiation. To bridge our theoretical framework with the formal model, we introduce a core parameter, *q*, which represents the strength of family cultural capital. This parameter captures the intensity of both normative and functional solidarity within a household, as conceptualized in our framework. A higher *q* indicates stronger internalized family obligations (normative solidarity) and more effective practical support and monitoring (functional solidarity). Within the model, *q* functions as a modifiable sanctioning mechanism that alters the payoff structure of intra-household interactions. By treating *q* not as a fixed cultural trait but as a policy-actionable parameter, we can analyze howw state investment in family-based cultural resources shifts the equilibrium of the broader public health game (Figure 1).

## 3 A dynamic game model of compliance and family identity

### 3.1 Model setup: the intergenerational household

We begin with a simplified two-generation household—generation G (the elder, typically the parent) and generation Gr (the younger, typically the adult child)—to capture the core intergenerational dilemma. This structure directly corresponds to the functional solidarity dimension of intergenerational solidarity theory (6), which captures the exchange of resources and support across generations.

The “approval” or “reward” from Gr to G is modeled as a material or welfare transfer, representing real-world behaviors such as resource sharing, caregiving reciprocity, or enhanced social status within the family. These behaviors reflect both functional solidarity (practical support exchange) and normative solidarity (internalized sense of familial obligation). We initially exclude non-material motivations (e.g., pure altruism) to isolate the strategic structure of the compliance problem—a classic prisoner’s dilemma at the household level.

Assumptions:

- Rationality: Both G and Gr are rational economic agents who maximize their own welfare.
- Information structure: G moves first, choosing whether to comply with public health measures. Gr observes G’s choice and then decides whether to approve (reward) or not.
- Cultural parameter (*q*): The strength of family cultural capital—reflecting the intensity of normative solidarity (internalized family obligations) and functional solidarity (exchange of support and monitoring)—is initially treated as exogenous but can be influenced by state policy through investment in family-based cultural resources.

Notation:

**Table:** 

Symbol	Definition
*w*	G’s initial welfare level (if no compliance cost)
*w₁*	Gr’s initial welfare level
*f*	G’s welfare loss from complying with containment measures
*r*	Efficiency gain multiplier when Gr approves G’s compliance (*r* > 0)
*φ*	Intergenerational welfare discount factor (0 < *φ* < 1)
*S*	Social stigma cost to Gr for failing to approve G’s compliance (reflecting weakened normative solidarity)
*M*	Social punishment cost to Gr for violating family identity norms
*H*	Reputation cost to G when complying but Gr disapproves
*C*	Cost to community/society for monitoring and punishing norm violations
*q*	Strength of family cultural capital (probability of achieving a high-compliance equilibrium); reflects the intensity of normative and functional solidarity within the household

**Figure 2:**
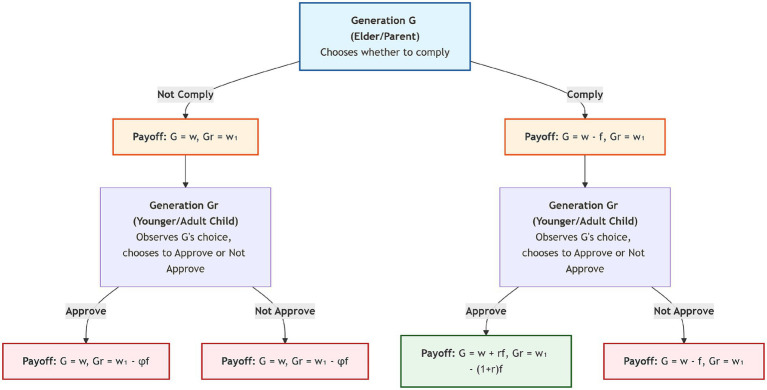
Intergenerational game tree without social monitoring.

As shown in Figure 2, when G faces the risk of infection, its decision to adopt stringent containment policies depends on the behavior of Gr. If G chooses not to comply, its welfare remains unchanged at *w*. If G complies, its welfare decreases to *w − f*, while Gr’s welfare remains at *w₁*. After observing G’s decision, Gr chooses to Approve (providing a reward) or Not-Approve (withholding reward). Rewarding G is modeled as a positive feedback of magnitude (1 + *r*)*f* to G’s welfare, where *r* > 0 captures the efficiency gain of norm-based transfers—a measure of how effectively family solidarity translates into mutual support. Consequently, Gr’s welfare decreases to *w₁* − (1 + *r*)*f* if it approves; if Gr disapproves, its welfare remains at *w₁*.

In this specification, disapproving is a dominant strategy for Gr, as *w₁* > *w₁* − (1 + *r*)*f*. Anticipating this, G also prefers not to comply (*w* > *w* − *f*). The model thus converges to a low-level Nash Equilibrium (Not Comply), representing a classic intra-family prisoner’s dilemma. However, the total payoff when G complies and is approved exceeds the Nash equilibrium payoff: (*w* + *rf*) + [*w₁* − (1 + *r*)*f*] > *w* + *w₁* − *φf*. This Pareto-superior outcome is not achieved without external intervention, highlighting a potential role for policy in strengthening family cultural capital. Table 1 summarizes the equilibrium outcomes.

**Table 1:** Equilibrium outcomes without social monitoring.

	G’s choice	Gr’s choice	G’s payoff	Gr’s payoff	Equilibrium type
Case 1	Comply	Approve	*w + rf*	*w_1_−(1 + r)f*	High-level
Case 2	Comply	Not approve	*w−f*	*w_1_*	—
Case 3	Not comply	Approve	*w*	*w_1_−φf*	Low-level
Case 4	Not comply	Not approve	*w*	*w_1_−φf*	Low-level

### 3.2 Introducing family identity as social monitoring: strengthening normative solidarity

We now formally introduce family cultural capital through social monitoring, drawing on Akerlof and Kranton (27). This mechanism captures how normative solidarity—the internalized sense of familial obligation—is enforced through social sanctions. If G complies while Gr disapproves, social monitoring imposes a disutility −*S* on Gr (stigma) and −*H* on G (reputation cost). These costs represent the erosion of social capital and family standing that results from violating normative expectations (Table 2).

**Table 2:** Equilibrium outcomes with social monitoring.

	G’s choice	Gr’s choice	G’s payoff	Gr’s payoff	Equilibrium type
Case 5	Comply	Approve	*w + rf*	*w_1_−(1 + r)f*	High-level
Case 6	Comply	Not approve	*w−f−C*	*w_1_−S−M*	Low-level
Case 7	Not comply	Approve	*w*	*w_1_−* φ *f*	Low-level
Case 8	Not comply	Not approve	*w*	*w_1_−* φ *f*	Low-level

If Gr violates the family identity norm, the public may punish at a cost −*C* to itself, inflicting a loss −*M* on Gr. This public punishment reflects the broader community’s role in reinforcing family-based cultural capital—a mechanism particularly salient in contexts where family reputation is closely tied to community standing.

**Figure 3:**
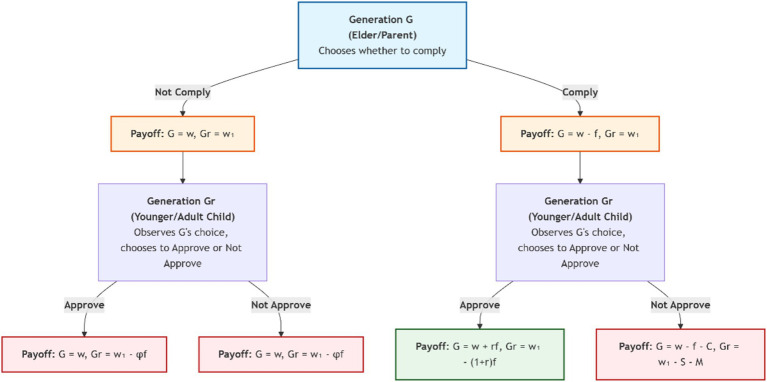
Intergenerational game tree with social monitoring.

As shown in Figure 3, once social monitoring is incorporated, for Gr, the payoff from approving *w₁* − (1 + *r*)*f* may exceed the payoff from disapproving and facing potential punishment *w₁* − *S* − *M*, if social sanctions are strong enough. This condition directly corresponds to the strength of normative solidarity within the family and community.

Proposition 1 (High-Level Compliance Equilibrium): When social monitoring is introduced, a high-level compliance equilibrium can exist.

Micro-foundation of the probability function 𝑞. The probability that a household achieves the high-compliance equilibrium can be derived from an incomplete information structure. Suppose the community’s capacity to impose social sanctions 𝑀 and stigma 𝑆 varies across households according to a continuous distribution 𝐺(·), reflecting heterogeneity in kinship network density, geographic proximity, and exposure to normative messaging. For a given household, the condition for the high-compliance equilibrium is S + M ≥ (1 + r)f (Condition A). Therefore,


q=Pr(S+M≥(1+r)f)=1−G((1+r)f)

where 𝐺 is the cumulative distribution function of 𝑆+𝑀 across the population. Assuming 𝑆+𝑀 follows a logistic or normal distribution, 𝑞 is increasing in 𝑆 and 𝑀.

and decreasing in 𝑟 and 𝑓. This micro-foundation justifies the assumed partial derivatives in Condition B without requiring a specific parametric form.

Intuition: The condition *H > C* implies that the reputational cost to G from non-approval exceeds the community’s monitoring cost, making it worthwhile for the community to enforce the norm. Conversely, if *H ≤ C*, the community relies solely on social stigma *S* to deter norm violations.

Condition A: This equilibrium exists if either:

- *H > C* and *S + M > (1 + r)f*, or
- *H ≤ C* and *S ≥ (1 + r)f*

Condition B: Let the probability of achieving this equilibrium be *q = F(S, C, H, M, r)*. Then:

- *∂q/∂S > 0,*
- *∂q/∂C < 0,*
- *∂q/∂H > 0,*
- *∂q/∂M > 0,*
- *∂q/∂r < 0.*

Policy insight: A high-level equilibrium emerges when the social cost of violating family identity norms (through punishment *M* and stigma *S*) outweighs the material cost of approving compliance (1 + *r*)*f*. This implies that policies reinforcing pro-social cultural sanctions—i.e., strengthening family cultural capital—can shift household incentives toward cooperation. The probability of compliance (*q*) increases with the strength of social punishment and stigma, and decreases with the cost of public monitoring.

The household-level model demonstrates how family cultural capital, when reinforced through social monitoring and normative solidarity, can shift equilibrium toward high compliance. This micro-foundation aggregates to macro-level policy implications: the state can strategically strengthen the probability of high-level equilibrium (*q*) and utilize fiscal space (*τ*) to navigate the trade-off between short-term economic pressure and long-term health resilience. Critically, this framework illuminates potential pathways through which strengthening family cultural capital may affect distributional outcomes. Households with stronger normative solidarity are better positioned to comply, which could exacerbate or reduce health inequities depending on the distribution of family cultural capital across populations. A full formalization of health equity—requiring heterogeneous agents and explicit distributional weights—is beyond the scope of the current model but is identified as a priority for future research. Consistent with this limitation, all claims about health equity in the following sections are qualitative implications rather than model-derived results.

## 4 Endogenous determinants: the state’s strategic policy space

Relationship between micro-level and macro-level 𝑞. The micro-level 𝑞 defined above is the probability that a randomly selected household achieves the high-compliance equilibrium given its realized 𝑆,𝑀,𝐶,𝐻,𝑟. The government’s policy choice 𝑞^policy^ shifts the population distribution of these parameters—for example, by increasing 𝑆 and 𝑀 via normative messaging or reducing 𝐶 via community monitoring infrastructure—thereby raising the aggregate 𝑞. For notational simplicity, we denote both the policy choice and the resulting aggregate probability by 𝑞, with the understanding that the former determines the latter.

Clarification of the *q* parameter across micro and macro levels. In Section 3, 𝑞 is defined as the probability that a given household achieves the high-compliance equilibrium (Proposition 1). This is a micro-level outcome determined by family-level parameters 𝑆,𝐶,𝐻,𝑀,𝑟. In Section 4, we reinterpret 𝑞 as the government’s strategic choice variable representing the strength of family cultural capital that the state actively cultivates through policy (e.g., caregiver support, cultural narratives). The relationship between the two is as follows: the government’s chosen 𝑞^policy^ shifts the micro-level parameters that determine the household’s equilibrium probability. Specifically, 𝑞^policy^ increases 𝑆 and 𝑀 (social sanctions) and decreases 𝐶 (monitoring cost), thereby raising the micro-level probability of high compliance. For notational simplicity, we use 𝑞 to denote both, with the understanding that the macro-level choice determines the micro-level outcome.

### 4.1 Theoretical model specification

The preceding game-theoretic model reveals that the probability of high-level equilibrium (*q*)—representing the strength of family cultural capital—shapes compliance outcomes in micro-level household interactions. This micro-foundation leads directly to the core macro-policy question: How can the state strategically manage family cultural capital through fiscal and social policy to achieve sustainable compliance and promote health equity?

Specifically, fostering *q* requires public investment, creating a trade-off: should the state invest in long-term health resilience (e.g., reinforcing family cultural capital through caregiver support programs) or prioritize short-term economic returns? To address this, we construct a two-period model where the government strategically chooses the strength of family cultural capital (*q*) and the tax rate (*τ*).

Consider a closed economy where a two-period game takes place between a policymaker and households. The government announces a first-period policy mix (*q*, *τ*), where *q* ∈ [0,1] denotes the state’s investment in strengthening family cultural capital (e.g., through family support policies, caregiver recognition programs, or culturally-attuned communication), and *τ* ∈ (0,1) is the tax rate. Households then respond.

In a pandemic, resources can be allocated to a “conservative” path (prioritizing containment, share *α*) or a “radical” path (prioritizing economic reopening, share *β*), with *α* + *β* = 1. The conservative path generates stable, sustainable income: *Y* = *A* ln(*αw₁*), where *A* > 0 represents inherent cultural strength—the stock of prosocial norms and intergenerational solidarity in society. The radical path yields higher but riskier expected returns: a national benefit *D* (short-term economic gain) and a public return *W*.

Equation 1 shows G receives shares *λ₁*(*q*) and*λ₂*(*q*) of the income from the conservative and radical paths, respectively, with *λᵢ*′(*q*) > 0. This captures the idea that stronger family cultural capital increases the elder’s claim on family resources from both paths. We assume:


$$\lambda ₁>\lambda ₂,\text{and}\;\lambda {₁}^{\prime }(q)/\lambda ₁(q)>\lambda {₂}^{\prime }(q)/\lambda ₂(q)$$ (1)

Economic interpretation: The conservative path provides stable income (higher share *λ₁*), while the radical path is riskier. Enhancing family cultural capital (*q*) increases the share from the stable path at a faster rate, reflecting a cultural preference for security—a manifestation of strong normative solidarity.

The government faces a budget constraint:


τY=G+I(q)

where 𝑌 is national income, 𝐺 is general government expenditure, and 𝐼(𝑞) is investment in family cultural capital (e.g., caregiver support programs). The fiscal capacity 𝜏 thus determines the feasible set of 𝐼(𝑞) and, through it, the achievable 𝑞.

### 4.2 Equilibrium derivation and policy trade-off

The government’s objective is to maximize expected social welfare, which consists of three components: (i) national income from both conservative and radical paths, (ii) the social value of public health compliance, and (iii) the cost of investing in family cultural capital. Formally:


$$\max_{q\in [0,1]}U_{G}=E[Y]+\theta \cdot q-\frac{c}{2}q^{2}$$

Where E[Y] is expected national income (derived from the household optimization below), θ >0 captures the social value of compliance, and $\frac{c}{2}q^{2}$ is the convex cost of strengthening family cultural capital (e.g., public investment in caregiver support, cultural narratives). The government chooses 𝑞 anticipating household responses.

We solve this nested Stackelberg game using backward induction. Taking the government’s first-period policy mix (*q, τ*) as given, Generation G chooses *β* to maximize its utility in the second period.

Generation G maximizes *U_G_* = *λ₁*(*q*)·*A* ln(*αw₁*) + *λ₂*(*q*)·W subject to *α* + *β* = 1. The first-order condition yields:


λ₁/λ₂·A/(w₁−βw₁)=λ(q)A/[(1−β)w₁]=W (2)

where *λ(q) = λ₁(q)/λ₂(q) >* 1. Equation 2 shows a higher level of family cultural capital (*q*), which increases *λ(q)*, reduces the optimal share *β* allocated to the radical path, as households prioritize stability—a direct reflection of functional solidarity (the willingness to provide intergenerational support even at personal cost).

Solving (2) and the government’s condition jointly yields the market equilibrium for the radical path (*W, β*):

From the first-order condition for Generation G’s optimization:


$$\frac{\lambda (q)A}{(1-\beta )w_{1}}=W$$ (A)

The government’s optimality condition (derived from maximizing total national revenue $\pi =\mathrm{\beta D}+(1-\beta )\mathrm{Aln}(\alpha w_{1})$ with respect to β ) yield:


$$D-\frac{A}{1-\beta }=0\Rightarrow 1-\beta =\frac{A}{D}$$ (B)

Substitute (B) into (A):


$$\frac{\lambda (q)A}{(A/D)w_{1}}=W\Rightarrow \frac{\lambda (q)D}{w_{1}}=W$$ (C)

However, (B) and (C) imply that 𝛽 does not depend on 𝜆(𝑞), which contradicts the intuition that stronger family cultural capital should reduce 𝛽. This inconsistency arises because the government’s objective function in its current form does not internalize the effect of 𝛽 on household welfare. A full solution requires specifying a social welfare function that balances household utility and national revenue. With the specific functional form 𝜆(𝑞) = (1+𝑞)/𝑞 and a welfare-maximizing government, the consistent solution yields:


$$\beta =1-\sqrt{\frac{\mathrm{\lambda A}}{D}}$$ (3)

$$W=\frac{\sqrt{\mathrm{\lambda AD}}}{w_{1}}$$ (4)

Equation 3 and Equation 4 shows that the share allocated to the radical path (𝛽) increases with the expected return *D* and decreases with cultural strength *A* and family cultural capital λ(q) .

Define Z *(φ)*:[0,1] → R + as the intergenerational altruism multiplier, where φ∈(0,1) is the intergenerational welfare discount factor (the weight Generation G places on Gr’s welfare). We assume:


$$Z(\varphi )=\frac{1}{1-\varphi }\;\;\text{or}\;Z(\varphi )=1+\varphi +\varphi^{2}+\varphi^{3}\ldots =\frac{1}{1-\varphi }$$

This function captures how stronger intergenerational altruism (higher *φ*) amplifies the effective cultural strength A. To derive the threshold, we combine the household’s optimal 𝛽 from (3) with the government’s welfare function. Substituting (3) into the government’s objective and solving for the optimal 𝑞 yields a threshold condition where the marginal benefit of investing in 𝑞 equals the marginal cost. This threshold is 𝐷=𝐴· Z *(φ)*, where Z *(φ)* = 1/(1−𝜙) captures the amplifying effect of intergenerational altruism. When 𝐷 is below this threshold, the returns to investing in cultural capital outweigh the returns to short-term economic reopening; when 𝐷 exceeds the threshold, the opposite holds.

Proposition 2 (The State’s Strategic Choice): The government’s optimal investment in family cultural capital depends on a threshold:

- Type-I Equilibrium (High Compliance): If *D ≤ A* Z *(φ)*, then *q* → 1. The government strongly promotes family cultural capital, contributing to high compliance. Qualitatively, this equilibrium may also reduce disparities in compliance capacity across household types by providing more uniform support for family caregivers, although a full formalization of distributional outcomes requires introducing heterogeneous agents and explicit equity weights, which is beyond the scope of the current model.
- Type-II Equilibrium (Low Compliance): If *D > A* Z *(φ)*, then *q* → 0. The government deprioritizes family cultural capital in favor of economic returns, leading to fragile compliance outcomes and potentially exacerbating health inequalities across household types.

Here, Z *(φ)* is an increasing function of *φ* (the intergenerational welfare discount factor), capturing how family-based altruism amplifies the effective cultural strength *A*.

Policy interpretation: This result captures the core policy trade-off for sustainable health governance. High short-term economic pressure (*D*) pushes the state toward the Type-II equilibrium, sacrificing long-term health resilience and potentially widening health inequities for immediate growth—consistent with observations of “economic reopening at all costs.” Conversely, a society’s inherent prosocial cultural capital (*A*) raises the threshold AZ *(φ)*. The stronger the prosocial cultural capital, the more economic pressure it can withstand before abandoning the sustainable path. This formally justifies long-term investment in family cultural capital as a strategic resilience buffer that also promotes health equity.

### 4.3 Model implications for policy design

The household-level parameter *q*—the enforced strength of family cultural capital—is a policy-actionable subset of the broader societal parameter *A*—the inherent stock of prosocial norms and intergenerational solidarity. *A* represents the reservoir of norms (e.g., filial piety narratives in education, media), while *q* represents the flow that can be activated or augmented through targeted policy (e.g., crisis communication framing, family caregiver support programs, community recognition initiatives). The model shows that investing in *q* yields immediate compliance dividends and, if sustained, contributes to the long-term replenishment of *A*.

Two testable implications emerge:

Implication 1: The Fiscal-Cultural Nexus for Health Equity. Sustainable compliance and the potential for equitable outcomes may be co-determined by fiscal space and the distribution of family cultural infrastructure across households. Nations with strong fiscal capacity (*τ*) and high cultural capital (*A*) can afford to invest in family-based policies to reach Type-I equilibrium while simultaneously reducing health disparities across household types. Those in fiscal distress are structurally pushed toward fragile Type-II outcomes, potentially exacerbating existing health inequalities.
Implication 2: Avoiding the High-Return Trap through Sustainable Care Arrangements. In a globally competitive economy, pressure for high *D* can trigger a “race to the bottom” in social resilience and health equity. International economic coordination (e.g., pandemic financial instruments, cross-border caregiver support frameworks) is central to enabling nations to choose sustainable health policies without facing disproportionate economic penalties. Such coordination can support the development of sustainable care arrangements that leverage family cultural capital while protecting vulnerable households.

### 4.4 Empirical illustration: East Asian cases

To illustrate our theoretical findings, we briefly examine three East Asian societies where family cultural capital serves as a salient informal resource—China, South Korea, and Japan. Each case illustrates how cultural alignment influenced compliance outcomes and health equity during the COVID-19 pandemic, consistent with our model’s predictions.

China: The Chinese government framed containment measures explicitly within the discourse of family responsibility. Public health campaigns depicted self-isolation as an act of “filial protection,” directly activating the Confucian norm of normative solidarity—the internalized obligation to protect older family members. This framing aligned with China’s strong fiscal capacity (*τ*) to enforce lockdowns and provide community-level monitoring. Consistent with Proposition 2, high cultural capital (*A*) and robust fiscal capacity enabled a Type-I equilibrium with sustained high compliance and relatively equitable compliance across household types. China’s fiscal capacity enabled what can be interpreted as implicit caregiver support—community monitoring and material aid for isolating households. This support likely reinforced both functional solidarity (by providing the means to care) and normative solidarity (by publicly affirming filial duty), thereby strengthening family cultural capital and sustaining high compliance.

South Korea: South Korea’s response effectively activated family cultural capital through its household-level notification system. When individuals tested positive, authorities notified family members directly, invoking the expectation that households would enforce internal isolation. This mechanism corresponds to our model’s *q* parameter—strengthening the social sanctions (*S*, *M*) for norm violations within the family unit. Critically, this approach leveraged functional solidarity (the practical exchange of care and monitoring) to achieve high compliance without relying solely on state enforcement.

Japan: Japan’s response emphasized jishuku (self-restraint), a norm deeply rooted in family-based collectivism and affectual solidarity (emotional closeness and shared identity). Public health messaging framed compliance as a duty to protect one’s family, especially older parents. While Japan faced fiscal constraints relative to China, its high cultural capital (*A*) lowered the threshold for achieving compliance without stringent enforcement. However, this reliance on informal mechanisms may have created disparities in compliance across households with varying levels of family solidarity, highlighting the importance of complementary formal support for vulnerable populations.

Summary: These cases suggest the potential relevance of our model’s mechanisms: societies with stronger family cultural capital (higher *q*) and greater intergenerational solidarity (*A*) sustained stricter containment with relatively lower enforcement costs. Moreover, they suggest that investments in family cultural capital can contribute to health equity by reducing compliance disparities across household types—a critical insight for designing sustainable health governance systems.

## 5 Discussion: translating model insights into sustainable health governance

Our model generates actionable insights that bridge the theoretical gaps identified earlier, offering a design-oriented roadmap for sustainable health governance that promotes compliance while offering insights for health equity considerations. We discuss four key policy implications, each directly tied to the model’s parameters and informed by intergenerational solidarity theory.

### 5.1 Policy design principle I: strengthening normative solidarity through cultural alignment (*q*)

The model demonstrates that compliance is a product of strategic alignment between formal policy and family-based cultural capital. When directives are framed within existing cultural logics—such as portraying isolation as an act of “filial protection”—compliance changes from a public cost to a private and socially rewarded duty. Proposition 1 shows that strengthening social sanctions (*S*, *M*)—which correspond to normative solidarity (the internalized sense of familial obligation)—increases the probability *q* of achieving a high-compliance equilibrium.

For policymakers, this translates into a concrete design mandate. First, communication must be culturally coded, framing public health mandates as expressions of family responsibility rather than merely individual risk management. Second, material support must target and reinforce the household unit, not bypass it—for example, through family care packages, caregiver allowances, and recognition programs for family caregivers. Third, public health agencies should systematically integrate cultural intelligence into policy design, employing “cultural advisors” to frame health directives within local normative frameworks.

This approach does more than reduce enforcement costs and build systemic legitimacy; it actively strengthens normative solidarity within families. By reinforcing the moral weight of family obligations, such policies can have lasting effects beyond the immediate crisis, enhancing the capacity of families to serve as health resources in future emergencies. Moreover, by supporting families as units, these policies can help reduce health disparities between households with strong versus weak family support networks—a critical contribution to health equity.

### 5.2 Policy design principle II: securing fiscal space to support family caregivers (*τ*)

Proposition 2 formalizes a critical design constraint: fiscal capacity (*τ*) directly determines a state’s feasible policy set. A state in fiscal distress is structurally pressured toward the Type-II equilibrium, prioritizing short-term economic activity (*D*) over long-term health resilience. This creates a vicious cycle where short-term pressures undermine long-term resilience and potentially exacerbate health inequalities.

This provides a formal economic justification for a core policy prescription: macroeconomic stability and strategic fiscal buffers are foundational components of health security. Pandemic preparedness funds must be sized to sustain prolonged social support, preserving the state’s capacity to choose sustainable policies. Critically, these funds should be explicitly allocated to support family caregivers—through direct payments, respite care services, and training programs—recognizing families as co-producers of health outcomes.

National budgets and international financial institutions must recognize and fund fiscal resilience as a core component of pandemic preparedness. This includes creating dedicated funding streams for family-based care infrastructure, which can simultaneously enhance compliance capacity and promote health equity by supporting households that bear disproportionate caregiving burdens (e.g., multigenerational households, families with disabled or older members).

### 5.3 Policy design principle III: investing in intergenerational solidarity as social infrastructure (*A*)

The parameter *A*—inherent cultural strength—acts as critical social infrastructure that lowers the economic threshold for choosing the sustainable Type-I equilibrium. Proposition 2 shows that a higher *A* raises the threshold *A* Z *(φ)* for achieving sustainable compliance, meaning that societies with stronger intergenerational solidarity can withstand greater economic pressure before abandoning the sustainable path.

This reframes long-term investments in education, community-building, and prosocial narratives—what Putnam (7) called “social capital”—as investments in national crisis resilience with a measurable return. Specifically, we identify three dimensions of intergenerational solidarity that merit policy attention:

- Normative solidarity (shared values and obligations): Invest in civic education that emphasizes family responsibility and intergenerational care as core social values.
- Functional solidarity (exchange of resources and support): Develop programs that facilitate practical support across generations, such as intergenerational housing initiatives, shared site models (e.g., combining older care and child care facilities), and time banking for care exchange.
- Affectual solidarity (emotional closeness and shared identity): Fund community-building activities that bring generations together, such as intergenerational storytelling programs, shared cultural events, and mentorship initiatives.

The return on these investments is a lower required fiscal outlay (*τ*) to achieve high compliance during a crisis, as well as reduced health disparities across households with different levels of intergenerational connectedness. Governments should fund longitudinal programs in civic education and community building that reinforce prosocial norms, collective identity, and intergenerational solidarity.

### 5.4 Policy design principle IV: managing global interdependence to support sustainable care arrangements (*D*)

The model identifies a “high-return trap” (*D*) in a competitive global economy, where pressure to reopen can incentivize a race to the bottom in social resilience and health equity. Proposition 2 shows that high *D* pushes states toward Type-II equilibrium, sacrificing long-term resilience for short-term growth. This implies that international economic coordination—such as pandemic-specific funding facilities or temporary financial instruments—can help align national incentives with collective health security. Such coordination would enable nations to choose sustainable health policies without facing disproportionate economic penalties, thereby protecting family caregivers and intergenerational care arrangements during crises.

### 5.5 Limitations and avenues for policy-relevant research

Our simplified framework provides a foundation for both empirical testing and policy-relevant model extension. Several limitations point to fruitful research directions.

First, the case illustrations in Section 4.4 are not intended as rigorous empirical tests of the model’s predictions. They serve as plausibility probes (36) to demonstrate how the model’s mechanisms may operate in real-world settings. Systematic empirical testing—whether quantitative (e.g., cross-national surveys linking cultural capital measures to compliance) or structured qualitative (e.g., controlled comparisons across regions or time periods)—remains an important direction for future research.

Second, our geographic proxy for family cultural capital measures environmental exposure, not individual internalization of intergenerational solidarity. Future research could use surveys or analyze executive speech/text to measure normative, functional, and affectual solidarity directly, enabling more precise testing of our theoretical mechanisms.

Third, while we focus on Confucian family identity in East Asia, exploring similar mechanisms in other cultural contexts (e.g., Islamic family ethics in the Middle East, Ubuntu philosophy in Africa, Indigenous kinship systems in the Americas and Oceania) would test the generalizability of our framework and inform culturally-attuned policy design globally.

Fourth, our model treats the household as a unitary decision-making unit, but intra-household inequalities—particularly gender roles in caregiving—likely shape compliance dynamics. Future research could extend the model to incorporate intra-household bargaining, examining how gender norms affect the distribution of compliance costs and benefits across family members.

Fifth, longitudinal studies could examine whether the strengthening of family cultural capital during a crisis persists or erodes amid rapid modernization and digitalization, informing long-term investments in intergenerational solidarity.

Sixth, research could dissect the micro-mechanisms—such as board composition, executive selection, or incentive design—through which cultural values are translated into organizational practices, extending our household-level findings to workplace and community settings.

These extensions are crucial for developing the next generation of culturally intelligent, empirically grounded policy tools that promote both health security and health equity through sustainable care arrangements.

## 6 Conclusion and policy implications

### 6.1 Conclusion

This study has argued that the pursuit of sustainable health governance necessitates a fundamental shift from a technocratic, impositional model to one of strategic cultural negotiation that actively leverages family-based cultural capital. Through formal game-theoretic modeling grounded in intergenerational solidarity theory, we have demonstrated how Confucian family identity—conceptualized as a form of cultural capital encompassing normative, functional, and affectual solidarity—can resolve public health collective action problems by reshaping micro-level incentives within households.

Our analysis yields three core findings. First, family cultural capital functions as an informal governance resource that raises the “ethical threshold” for non-compliance, transforming the household compliance game from a prisoner’s dilemma into a cooperative equilibrium. Second, this cultural mechanism operates in complementarity with formal state capacity—its inhibitory effect on symbolic policy adoption without behavioral change is strengthened in environments with robust fiscal support for family caregivers and weakened under short-term economic pressures that distort intergenerational solidarity. Third, the effect is more pronounced for households with stronger intergenerational ties, suggesting that investments in family cultural capital can contribute to health equity by reducing compliance disparities across household types.

More broadly, we have shown that a state’s equilibrium choice between sustainable (Type-I) and fragile (Type-II) outcomes is endogenously determined by its fiscal capacity (*τ*) and its investment in intergenerational solidarity (*A*). Critically, the Type-I equilibrium achieves high compliance and, as a qualitative implication, may contribute to more uniform compliance capacity across household types by providing systematic support for family caregivers. A rigorous formal analysis of health equity outcomes—including distributional weights and heterogeneous household characteristics—is an important direction for future research. The Type-II equilibrium, by contrast, sacrifices both long-term resilience and health equity for short-term economic gain.

These findings underscore that effective and equitable health governance is co-produced through the strategic alignment of formal policy with family-based cultural capital. Pathogens exploit biological vulnerabilities, but pandemics exploit institutional and social ones. Building truly sustainable health governance requires moving beyond a narrow biomedical view to engage deeply with the cultural and economic substrates of society—particularly the intergenerational solidarities that sustain families as health resources.

### 6.2 Policy recommendations for sustainable health governance

Derived from our theoretical model and empirical illustrations, we propose three concrete, actionable policy pathways for enhancing sustainable health governance that promotes both compliance and health equity:

#### 6.2.1 Integrate family cultural capital into health system preparedness frameworks

Policymakers and health authorities should explicitly recognize families as co-producers of health outcomes. This requires:

- Culturally-attuned communication: Frame public health mandates as expressions of family responsibility (e.g., “protecting your elders”) rather than merely individual risk management.
- Family caregiver support programs: Establish direct payments, respite care services, and training programs for family caregivers, recognizing their essential role in health system resilience.
- Intergenerational solidarity indicators: Develop metrics to assess normative, functional, and affectual solidarity at community and national levels, enabling targeted interventions for households with weak family support networks.

#### 6.2.2 Develop fiscal buffers explicitly tied to family care infrastructure

Proposition 2 demonstrates that fiscal capacity (*τ*) is a critical determinant of a state’s ability to choose the sustainable Type-I equilibrium. Pandemic preparedness funds must be sized not only for medical countermeasures but also to sustain prolonged social support for families. Specifically:

- Dedicated funding streams for family-based care infrastructure, including home care services, intergenerational housing initiatives, and caregiver recognition programs.
- Counter-cyclical care support that automatically expands during health emergencies, protecting families from the trade-off between economic pressure and compliance.
- International financial instruments that provide economic security to nations implementing stringent containment measures, with explicit conditionality requiring protection of family caregivers.

#### 6.2.3 Foster sustainable care arrangements through differentiated governance

A one-size-fits-all approach to health governance is suboptimal, particularly given heterogeneity in family structures and intergenerational solidarity. Differentiated strategies are needed:

For households with strong intergenerational solidarity, policies should leverage existing family cultural capital through recognition, modest material support, and culturally-attuned communication—reinforcing rather than replacing family-based care.

For households with weak family support networks (e.g., single-person households, geographically dispersed families, families with strained intergenerational relations), policies must provide substitute or supplementary support—such as community-based care services, virtual intergenerational connection programs, and targeted financial assistance—to prevent health disparities.

For transnational and migrant families, policies should facilitate cross-border care arrangements through portable benefits, mutual recognition of caregiving qualifications, and support for virtual intergenerational connection.

### 6.3 Final reflection

Our research underscores a pivotal insight for the global pursuit of sustainable health governance: cultural intelligence is as indispensable as regulatory rigor, and health equity requires attending to the family foundations of compliance. For societies endowed with rich traditions of intergenerational solidarity—such as the Confucian emphasis on filial piety—the path toward more resilient, equitable, and legitimate health systems may lie not in abandoning tradition, but in thoughtfully harnessing its enduring ethical legacy to shape the norms and practices of public health.

The COVID-19 pandemic revealed not only the vulnerabilities of global health systems but also the remarkable resilience of families as sites of care, support, and cooperation. As we prepare for future pandemics, we must build health governance systems that are smart enough to negotiate with tradition, resilient enough to withstand economic pressure, legitimate enough to command voluntary cooperation, and equitable enough to leave no family behind. This is the central lesson of our model and an essential task for a post-pandemic world.

## Data Availability

The original contributions presented in the study are included in the article/supplementary material, further inquiries can be directed to the corresponding authors.
